# LRRK2 Kinase Inhibition Attenuates Neuroinflammation and Cytotoxicity in Animal Models of Alzheimer’s and Parkinson’s Disease-Related Neuroinflammation

**DOI:** 10.3390/cells12131799

**Published:** 2023-07-06

**Authors:** Veronica Mutti, Giulia Carini, Alice Filippini, Stefania Castrezzati, Lorena Giugno, Massimo Gennarelli, Isabella Russo

**Affiliations:** 1 IRCCS Istituto Centro San Giovanni di Dio Fatebenefratelli, 25125 Brescia, Italy; vmutti@fatebenefratelli.eu (V.M.);; 2 Biology and Genetics Unit, Department of Molecular and Translational Medicine, University of Brescia, 25123 Brescia, Italy; 3 Human Anatomy Unit, Department of Biomedical Sciences and Biotechnologies, University of Brescia, 25123 Brescia, Italy

**Keywords:** LRRK2, inhibitor, neuroinflammation, Parkinson’s disease, Alzheimer’s disease

## Abstract

Chronic neuroinflammation plays a crucial role in the progression of several neurodegenerative diseases (NDDs), including Parkinson’s disease (PD) and Alzheimer’s disease (AD). Intriguingly, in the last decade, leucine-rich repeat kinase-2 (*LRRK2*), a gene mutated in familial and sporadic PD, was revealed as a key mediator of neuroinflammation. Therefore, the anti-inflammatory properties of LRRK2 inhibitors have started to be considered as a disease-modifying treatment for PD; however, to date, there is little evidence on the beneficial effects of targeting LRRK2-related neuroinflammation in preclinical models. In this study, we further validated LRRK2 kinase modulation as a pharmacological intervention in preclinical models of AD- and PD-related neuroinflammation. Specifically, we reported that LRRK2 kinase inhibition with MLi2 and PF-06447475 (PF) molecules attenuated neuroinflammation, gliosis and cytotoxicity in mice with intracerebral injection of Aβ_1-42_ fibrils or α-syn preformed fibrils (pffs). Moreover, for the first time in vivo, we showed that LRRK2 kinase activity participates in AD-related neuroinflammation and therefore might contribute to AD pathogenesis. Overall, our findings added evidence on the anti-inflammatory effects of LRRK2 kinase inhibition in preclinical models and indicate that targeting LRRK2 activity could be a disease-modifying treatment for NDDs with an inflammatory component.

## 1. Introduction

Neuroinflammatory response plays a crucial role in the defense mechanisms of the brain [1]. However, neuroinflammation may act as a “double-edged sword” because the release of excessive quantities of proinflammatory mediators could lead to neuronal damage and degeneration [2]. Indeed, it is well established that immunological processes contribute to the pathogenesis and disease symptoms of several neurodegenerative diseases (NDDs), including Parkinson’s disease (PD) and Alzheimer’s disease (AD) [3,4]. In this regard, accumulating evidence has revealed the key involvement of immune cells in neuron damage and loss [2]. The brain immune system consists of resident microglia and astrocytes, with the contribution of external immune cells, like T-cells and neutrophils, which infiltrate the brain following disruption of the blood–brain barrier (BBB) [5]. In numerous NDDs, immune cells have been reported to be chronically activated, even by the presence of amyloid protein aggregates, and adopt a reactive phenotype that contributes to neuronal dysfunctions and degeneration [2]. Taken together, these observations indicate that although a well-regulated inflammatory reaction is essential for tissue repair, a protracted immune response can result in a severe and chronic neuroinflammatory cycle that promotes the progression of neurodegeneration and disease [6]. Therefore, it is time to speculate that a dynamic modulation of neuroinflammation might represent a disease-modifying therapeutic strategy for NDDs.

Of relevance, leucine-rich repeat kinase 2 (LRRK2), a kinase linked to genetic and sporadic PD [7,8,9], has been revealed as a positive mediator of neuroinflammatory response [10,11,12,13,14,15,16]. *LRRK2* encodes a complex multidomain protein characterized by an enzymatic core with GTPase and serine/threonine kinase activities [17]. PD-segregating mutations reside in the catalytic core of the protein and can affect either the kinase (G2019S and I2020T) or the GTPase (N1347H, R1441C/G/H and Y1699C) activities [7], suggesting that targeting the enzymatic activity of the protein could be beneficial. Regarding the link between LRRK2 and inflammation, in the last decade, it has been widely shown that the kinase activity of LRRK2 controls the generation of proinflammatory molecules in brain immune cells, microglia [14,18,19,20] and astrocytes [11,19,21]. This LRRK2-related function has also been corroborated using in vivo studies. Indeed, transgenic mice with LRRK2 G2019S mutation, which increases the kinase activity of the protein by about threefold, exhibited increased gliosis and neuroinflammation under PD-related conditions [22,23,24], while LRRK2 KO rodents displayed mitigated neuroinflammatory effects upon different inflammatory challenges [13,25,26]. Taken together, these in vitro and in vivo findings clearly define LRRK2 kinase activity as a positive modulator of the brain immune response.

Although the anti-inflammatory properties of LRRK2 inhibitors are starting to be considered as disease-modifying treatment for PD, little is known about the beneficial effects of targeting LRRK2-related neuroinflammation in preclinical models. In this regard, a few studies reported that LRRK2 kinase inhibition leads to an attenuation of neuroinflammation and neurodegeneration under pathological conditions [23,27,28,29], thus supporting the idea that lowering LRRK2 kinase activity has an anti-inflammatory effect and could be neuroprotective during the pathology. In this study, we further validated the modulation of LRRK2 kinase activity as a pharmacological intervention in diseased brains. To this aim, we explored the effects of two different LRRK2 inhibitors in animal models of AD- and PD-related neuroinflammation. Specifically, we found that LRRK2 kinase inhibition with MLi2 and PF-06447475 (PF) attenuated neuroinflammation, gliosis and cell toxicity in mice with intracerebral injection of Aβ_1-42_ fibrils (our AD mouse model) or of α-syn preformed fibrils (pffs; our PD mouse model). Overall, our findings further indicate that targeting LRRK2 activity could be a disease-modifying treatment for NDDs with an inflammatory component.

## 2. Materials and Methods

### 2.1. Aβ_1-42_ Fibrils and α-syn pff Generation and Characterization

Aβ_1-42_ fibrils were generated as we recently reported [11]. In detail, human Aβ_1-42_ (Bachem, Bubendorf, Switzerland) was resuspended in cold hexafluoroisopropanol (HFIP, Merck, Darmstadt, Germany/Sigma-Aldrich, St. Louis, MO, USA) and maintained under rotation at room temperature (RT) overnight (ON). Aβ_1-42_ solution was then aliquoted, speed-vacuum dried, and stored at −80 °C until use. To remove possible protein aggregation, before injection Aβ_1-42_ was dissolved in anhydrous dimethylsulfoxide (DMSO, Merck/Sigma-Aldrich) and sonicated for 10 min at RT. Then, Aβ_1-42_ was resuspended in PBS and incubated at 37 °C for 48 h to obtain a fibril-enriched preparation, while human α-syn pffs were generated as we previously reported [30]. Human monomeric α-syn (Proteos, Kalamazoo, MI, USA) was dissolved in PBS at 5 mg/mL and incubated at 37 °C for 7 days under constant shaking to induce aggregation. Enriched pffs were isolated from the soluble part of the preparation using centrifugation at 14,000 rpm for 15 min, and then quantified in relation to the initial concentration of monomer before fibrillation, as previously described [30]. Before injection, α-syn pffs diluted at 2.5 mg/mL in PBS were sonicated for 5 s on and 5 s off for a total of 30 s by using a 50/60 Hz ultrasonic bath (J.P. Selecta, Barcelona, Spain).

Aβ_1-42_ fibrils and α-syn pff fibrillizations were verified using ThioflavinT (ThioT, Merck/Sigma Aldrich) assays and transmission electron microscopy (TEM). Briefly, 7 µg of fibril was incubated with 5 µM of ThioT for 1 min at RT. Control measurement was performed with 5 µM ThioT in PBS for detection of background fluorescence intensity. We detected fluorescence emission at 482 nm with excitation at 450 nm by using the PerkinElmer^®^ EnSight—Multimode Plate Reader. For TEM, 100 ng of Aβ_1-42_ fibril and α-syn pff before and after sonication were incubated on a 400 mesh formvar-coated grid (TAAB Ltd., Singapore) for 2 min at RT. After removing the excess of solution from the grid, samples were negatively stained with Uranyless (Electron Microscopy Sciences, Hatfield, PA, USA) for 2 min at RT and examined using TEM (Tecnai G2 Spirit; FEI Company, Eindhoven, The Netherlands) at 80 kV.

### 2.2. Animals: Stereotaxic Surgery and LRRK2 Inhibitor Administration

Animal procedures were performed in accordance with European Community Directive 2010/63/UE and approved by the Ethics Committee of the University of Brescia (Project ID: 708-2018-PR). Three-month-old C57BL/6J mice were purchased from Charles River and maintained under regular lighting conditions (12 h light–dark cycle) with free access to food and water. Before all the experimental procedures, the mice were kept in an animal facility for at least 10 days.

Before intracerebral injections, mice received LRRK2 inhibitor PF (10 mg/kg, twice daily intraperitoneal injection (ip)), MLi2 (10 mg/kg, twice daily ip) or vehicle (twice daily, ip) for 10 consecutive days. Both inhibitors were dissolved in 30% hydroxypropyl-β-cyclodextrin (Sigma Aldrich, St. Louis, MO, USA) in saline solution. Three days after the initiation of drug administration, Aβ_1-42_ fibrils, Aβ_1-42_ monomer (Mon), α-syn pffs or α-syn Mon were intracerebrally injected. Specifically, mice were treated with Rimadyl (5 mg/kg; subcutaneous injection) and after 30 min were anesthetized with a mixture of Zoletil and Xylazine (30 mg/kg–10 mg/kg; ip). Mice were then placed into a stereotaxic frame where an incision was made above the midline and their skulls were exposed using cotton tips. Aβ_1-42_ fibrils or Aβ_1-42_ Mon (2.25 μg in 5 μL of PBS) were injected intracerebroventricularly (icv) into the lateral ventricle using a 10 μL syringe (World Precision Instruments, Sarasota, FL, USA) at a rate of 1 μL/min. The coordinates for the stereotaxic infusion were −2.5 mm dorsal/ventral, −/+1 mm lateral and +0.4 mm anterior/posterior from the bregma (bilateral injection). α-Syn pffs or α-syn Mon (5 μg in 2 μL of PBS) were injected into the dorsal striatum using a 10 μL syringe (World Precision Instruments, Sarasota, FL, USA) at a rate of 1 μL/min. The coordinates for the stereotaxic infusion were −3.2 mm dorsal/ventral, −/+2 mm lateral and +0.2 mm anterior/posterior from the bregma (bilateral injection). The needle was left in place for an additional 5 min before being slowly retracted from the brain. Mice were then sacrificed 1 week after the intracerebral injections. The left hemisphere was postfixed for 10 days in 4% paraformaldehyde for immunohistochemical examination, and the right hemisphere was homogenized for biochemical analysis.

### 2.3. Immunohistochemistry

For immunohistochemistry (IHC), we used the peroxidase method (Vector Laboratories, Vectastain Elite, Burlingame, CA, USA). Specifically, free-floating sections were treated with 3% H_2_O_2_ in water for 5 min to block endogenous peroxidase. After washing in PBS, the sections were incubated in blocking solution (0.1% Triton X-100/5% serum in PBS) for 30 min and then incubated with primary antibodies in the same solution ON at 4 °C. As primary antibody, we used mouse anti-iNOS (Santa Cruz, Santa Cruz, CA, USA, Sc7271, 1:100), rabbit anti-Iba1 (Wako, 018-19741, Osaka, Japan 1:300) and rat anti-GFAP (Invitrogen, 13-0300, 1:500). After several washes with PBS, sections were incubated for 1 h with biotinylated secondary antibody at RT. Then, an avidin–biotin–peroxidase complex was applied for 30 min followed by peroxidase detection for 2 min (DAB, 3,3′-diaminobenzidine tetrahydrochloride, enhanced liquid substrates system, 1:30; Sigma Aldrich, St. Louis, MO, USA). Sections were mounted on Super Frost slides (Thermo Fisher Scientific, Waltham, MA, USA) and completely dried, then dehydrated with graded concentrations of alcohol (50, 70, 90 and 100%; 1 min each) and immersed in xylene before being cover-slipped with DPX mounting media. The slices were visualized and acquired using a light microscope Olympus BX50 and a 20× objective (Olympus, Shinjuku, Japan).

For IHC staining quantification, we evaluated the number of cells, intensity density (Integrated Density) and the area occupied by the signal (% of the area) for Iba1 and GFAP markers and the intensity density and the area occupied by the signal (% of the area) for iNOS. In detail, we analyzed 5 sections (1 every 6 through the hippocampus for Aβ_1-42_ -injected mice and 1 every 6 through the striatum for α-syn-injected mice) for each mouse, and the results are shown as the average of all the sections analyzed for each mouse.

### 2.4. Brain Lysis and Western Blotting

After brain dissection (cortex, hippocampus and striatum), tissues were frozen by immersion in liquid nitrogen. After homogenization, total proteins were extracted with cold lysis buffer (20 mM Tris–HCl pH 7.5, 150 mM NaCl, 1 mM EDTA, 2.5 mM sodium pyrophosphate, 1 mM β-glycerophosphate, 1 mM Na3VO4, 1% Triton-X-100, protease inhibitors), incubated on ice for 30 min and centrifuged at 14,000 rpm at 4 °C for 30 min. Total protein concentration was measured by using the PierceTM BCA protein assay (Thermo Fisher Scientific). Then, 50 μg of total protein was separated using electrophoresis on 7.5% polyacrylamide gels or Criterion Tris-HCl precast gels (Bio-Rad, Hercules, CA, USA) and then transferred to PVDF membrane (Bio-Rad). After saturation with 5% nonfat dry milk, membranes were incubated ON at 4 °C with the following primary antibodies: rabbit anti-LRRK2 phospho Ser935 (Abcam, Cambridge, UK, ab133450, 1:300) rabbit anti-LRRK2 (Abcam ab133474, 1:300), goat anti-IL-1β (R&D System, Minneapolis, MN, USA, AF-401-NA, 1:500), mouse anti-CASP-3 (SantaCruz, Dallas, TX, USA, sc-7272 1:300), rabbit anti-Cyclooxygenase-2 (COX-2, Cayman,160106 1:500) and mouse anti-GAPDH (Thermo Fisher Scientific, MA5-15738, 1:30.000). Subsequently, membranes were incubated for 1 h at RT with HRP-conjugated secondary antibodies (Merck-Sigma Aldrich) and finally with ECL Western blot substrate (GE Healthcare, Chicago, IL, USA).

### 2.5. Statistical Analysis

All data were expressed as mean ± SEM and represent at least three animals per group. Specifically, Aβ_1-42_ Mon: n = 3; Aβ_1-42_ Fibr: n = 4; Aβ_1-42_ Fibr—MLi2: n = 4; Aβ_1-42_ Fibr—PF: n = 4; α-syn Mon: n = 4; α-syn pffs: n = 4; α-syn pffs—MLi2: n = 4; α-syn pffs—PF: n = 4. Statistical significance of differences between groups was assessed using one-way ANOVA followed by Bonferroni’s post-hoc test. Data were analyzed using Prism software (v8.0; GraphPad Software Inc., San Diego, CA, USA) and statistical significance was taken at *p* < 0.05.

## 3. Results

### 3.1. Generation of Aβ_1-42_ Fibrils and α-syn pffs for Intracerebral Injection

**Figure 1:**
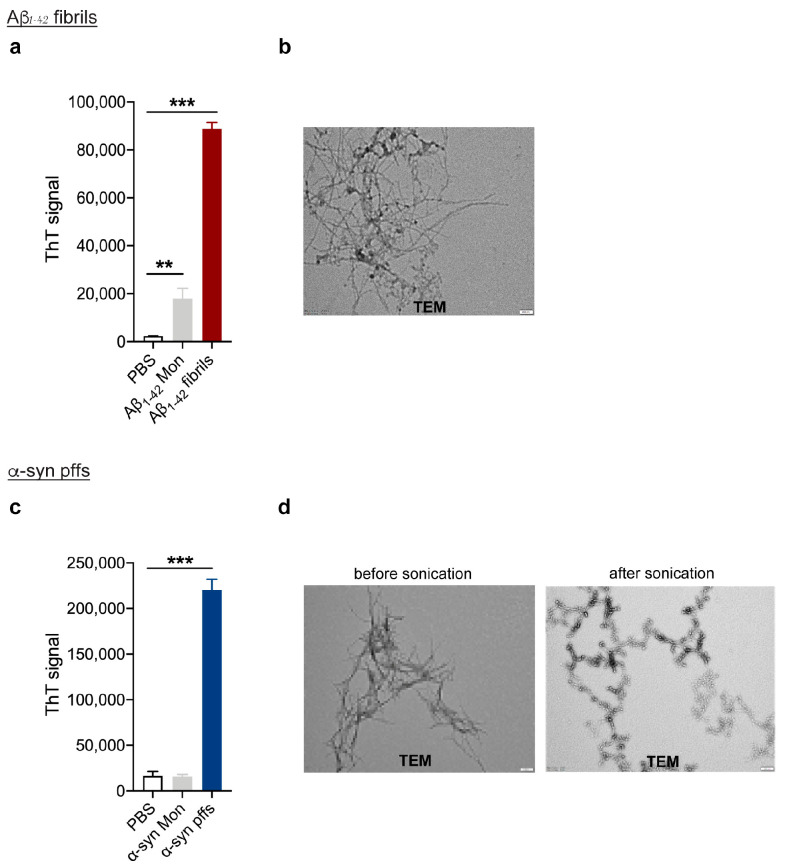
Aβ_1-42_ fibril and α-syn pff generation and characterization. (**a**) ThioT assay shows a greater amount of fluorescent signal in Aβ_1-42_ fibril-enriched preparation compared to monomeric protein and control PBS. Data are representative of at least three preparations and are expressed as the mean ± SEM. Data were analyzed using one-way ANOVA with Bonferroni’s post-hoc test: Aβ_1-42_ Mon vs. PBS, ** *p* = 0.0017; Aβ_1-42_ Mon vs. Aβ_1-42_ fibrils, *** *p* < 0.0001; Aβ_1-42_ fibrils vs. PBS, *** *p* < 0.0001. (**b**) TEM performed on Aβ_1-42_ preparation reveals thread-like fibril structure. Scale bar 200 nm. (**c**) ThioT assay shows a greater amount of fluorescent signal in α-syn fibril-enriched preparation compared to monomeric protein and control PBS. Data are representative of at least three preparations and are expressed as mean ± SEM. Data were analyzed using one-way ANOVA with Bonferroni’s post-hoc test: α-syn Mon vs. PBS; α-syn Mon vs. α-syn pffs, *** *p* < 0.0001; α-syn pffs vs. PBS, *** *p* < 0.0001. (**d**) TEM performed on α-syn pffs preparation before and after sonication. Scale bar 200 nm.

In order to corroborate the anti-inflammatory effects of LRRK2 inhibition in preclinical models of AD- and PD-related neuroinflammation, we first generated and validated Aβ_1-42_ and α-syn fibrils for intracerebral injection (Figure 1). Aβ_1-42_ fibrils were prepared from human Aβ_1-42_ monomeric protein incubated for 48 h at 37 °C to induce aggregation, while α-syn pffs were generated from human monomeric α-syn incubated for 7 days at 37 °C. The formation of fibrils was verified using two different approaches; specifically, a ThioT assay that detected a greater amount of fluorescence signal in fibrils preparation compared to control solvent or monomeric protein (Figure 1a,c) and TEM examination that reported thread-like fibrillar structures (Figure 1b,d). Taken together, these results indicate a good quality of our Aβ_1-42_ and α-syn fibril-enriched preparation.

### 3.2. LRRK2 Inhibition Attenuates Gliosis in Animal Models of AD- and PD-Related Neuroinflammation

**Figure 2:**
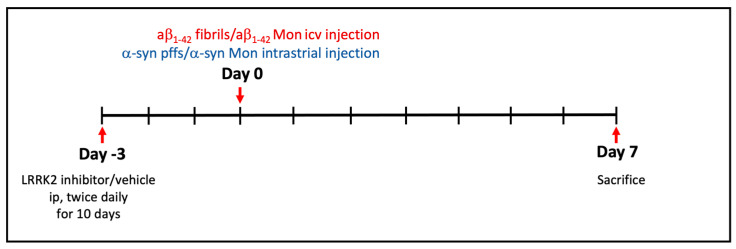
Schematic representation of the animal procedures performed during the experiment. LRRK2 inhibitor (MLi2/PF; 10 mg/kg; ip)/vehicle was administrated twice daily for 10 consecutive days. Three days after the initiation of LRRK2 inhibitor administration, Aβ_1-42_ fibrils, Aβ_1-42_ Mon, α-syn pffs or α-syn Mon were intracerebrally injected (bilateral injection). Mice were then sacrificed 7 days after the intracerebral injections, half hemisphere was processed for IHC analysis and half for protein lysates.

To further demonstrate the ability of LRRK2 pharmacological inhibition to mitigate neuroinflammatory effects in vivo, we investigated the neuroinflammation and toxicity caused by intracerebral injection of AD- and PD-related aggregates in mice after intraperitoneal administration of two different LRRK2 inhibitors (as reported in the schematic Figure 2). To this aim, we first confirmed that LRRK2 kinase inhibition occurs in mice following MLi2 and PF inhibitor administration by analyzing phosphorylation of Ser935-LRRK2, the most used pharmacodynamic biomarker of LRRK2 kinase inhibitors [31,32]. As predicted, we observed reduced levels of Ser935-LRRK2 phosphorylation in the cortex region of both animal models (Aβ_1-42_ fibril- and α-syn pff-injected mice) when treated with MLi2 and PF inhibitors (Figure S1), indicating LRRK2 kinase inhibition. Moreover, in support of LRRK2 as a stress-response kinase upon inflammatory challenges [13,15,33,34], we found that both Aβ_1-42_ fibril and α-syn pff injection induced increased levels of pSer935-LRRK2 compared to their respective control mice (Figure S1). These findings are in accordance with our previous in vitro results, where we reported augmented phosphorylation of Ser935-LRRK2 in microglia and astrocyte primary cultures upon treatment with α-syn pffs or with Aβ_1-42_ fibrils, respectively [11,15]. Taken together, these observations indicate that, both in cultured cells and in preclinical models, LRRK2 is phosphorylated and recruited in the cellular pathways activated by AD- and PD-related aggregates.

**Figure 3:**
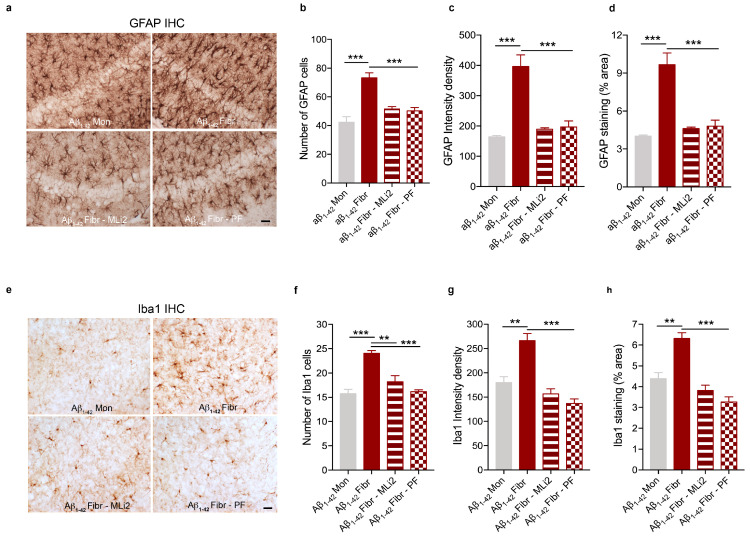
LRRK2 inhibition attenuates gliosis in response to Aβ_1-42_ fibril injection. (**a**) Representative images of GFAP staining in the hippocampus of mice injected with Aβ_1-42_ Mon, Aβ_1-42_ fibrils, Aβ_1-42_ fibrils with MLi2 inhibitor, and Aβ_1-42_ fibrils with PF inhibitor. Scale bar 30 μm. (**b**) Quantification of the number of GFAP cells. Data are representative of at least three animals per group and are expressed as mean ± SEM. Data were analyzed using one-way ANOVA with Bonferroni’s post-hoc test: Aβ_1-42_ Mon vs. Aβ_1-42_ fibrils, *** *p* < 0.0001; Aβ_1-42_ fibrils vs. Aβ_1-42_ fibrils with MLi2, *** *p* = 0.0010; Aβ_1-42_ fibrils vs. Aβ_1-42_ fibrils with PF, *** *p* = 0.0006. (**c**) Quantification of GFAP immunoreactivity expressed as intensity density. Data are representative of at least three animals per group and are expressed as mean ± SEM. Data were analyzed using one-way ANOVA with Bonferroni’s post-hoc test: Aβ_1-42_ Mon vs. Aβ_1-42_ fibrils, *** *p* = 0.0002; Aβ_1-42_ fibrils vs. Aβ_1-42_ fibrils with MLi2, *** *p* = 0.0003; Aβ_1-42_ fibrils vs. Aβ_1-42_ fibrils with PF, *** *p* = 0.0004. (**d**) Quantification of GFAP immunoreactivity expressed as % area occupied by the signal. Data are representative of at least three animals per group and are expressed as mean ± SEM. Data were analyzed using one-way ANOVA with Bonferroni’s post-hoc test: Aβ_1-42_ Mon vs. Aβ_1-42_ fibrils, *** *p* = 0.0002; Aβ_1-42_ fibrils vs. Aβ_1-42_ fibrils with MLi2, *** *p* = 0.0003; Aβ_1-42_ fibrils vs. Aβ_1-42_ fibrils with PF, *** *p* = 0.0004. (**e**) Representative images of Iba1 staining in the hippocampus of mice injected with Aβ_1-42_ Mon, Aβ_1-42_ fibrils, Aβ_1-42_ fibrils with MLi2 inhibitor, and Aβ_1-42_ fibrils with PF inhibitor. Scale bar 30 μm. (**f**) Quantification of the number of Iba1 cells. Data are representative of at least three animals per group and are expressed as mean ± SEM. Data were analyzed using one-way ANOVA with Bonferroni’s post-hoc test: Aβ_1-42_ Mon vs. Aβ_1-42_ fibrils, *** *p* = 0.0002; Aβ_1-42_ fibrils vs. Aβ_1-42_ fibrils with MLi2, ** *p* = 0.0021; Aβ_1-42_ fibrils vs. Aβ_1-42_ fibrils with PF, *** *p* = 0.0002. (**g**) Quantification of Iba1 immunoreactivity expressed as intensity density. Data are representative of at least three animals per group and are expressed as mean ± SEM. Data were analyzed using one-way ANOVA with Bonferroni’s post-hoc test: Aβ_1-42_ Mon vs. Aβ_1-42_ fibrils, ** *p* = 0.0025; Aβ_1-42_ fibrils vs. Aβ_1-42_ fibrils with MLi2, *** *p* = 0.0002; Aβ_1-42_ fibrils vs. Aβ_1-42_ fibrils with PF, *** *p* < 0.0001. (**h**) Quantification of Iba1 immunoreactivity expressed as % area occupied by the signal. Data are representative of at least three animals per group and are expressed as mean ± SEM. Data were analyzed using one-way ANOVA with Bonferroni’s post-hoc test: Aβ_1-42_ Mon vs. Aβ_1-42_ fibrils, ** *p* = 0.0029; Aβ_1-42_ fibrils vs. Aβ_1-42_ fibrils with MLi2, *** *p* = 0.0002; Aβ_1-42_ fibrils vs. Aβ_1-42_ fibrils with PF, *** *p* < 0.0001.

**Figure 4:**
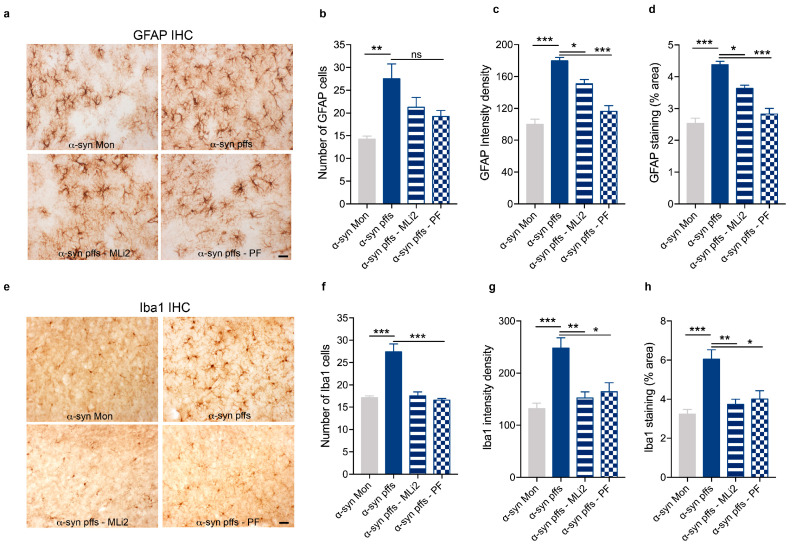
LRRK2 inhibition attenuates gliosis in response to α-syn pffs injection. (**a**) Representative images of GFAP staining in the striatum of mice injected with α-syn Mon, α-syn pffs, α-syn pffs with MLi2, and α-syn pffs with PF inhibitor. Scale bar 30 μm. (**b**) Quantification of the number of GFAP cells. Data are representative of four animals per group and are expressed as mean ± SEM. Data were analyzed using one-way ANOVA with Bonferroni’s post-hoc test: α-syn Mon vs. α-syn pffs, ** *p* = 0.0046, α-syn pffs vs α-syn pffs with PF not significant (ns) (**c**) Quantification of GFAP immunoreactivity expressed as intensity density. Data are representative of four animals per group and are expressed as the mean ± SEM. Data were analyzed using one-way ANOVA with Bonferroni’s post-hoc test: α-syn Mon vs. α-syn pffs, *** *p* < 0.0001; α-syn pffs vs. α-syn pffs with MLi2, * *p* = 0.0275; α-syn pffs vs. α-syn pffs with PF, *** *p* < 0.0001. (**d**) Quantification of GFAP immunoreactivity expressed as % area occupied by the signal. Data are representative of four animals per group and are expressed as the mean ± SEM. Data were analyzed using one-way ANOVA with Bonferroni’s post-hoc test: α-syn Mon vs. α-syn pffs, *** *p* < 0.0001; α-syn pffs vs. α-syn pffs with MLi2, * *p* = 0.0162; α-syn pffs vs. α-syn pffs with PF, *** *p* < 0.0001. (**e**) Representative images of Iba1 staining in the striatum of mice injected with α-syn Mon, α-syn pffs, α-syn pffs with MLi2, and α-syn pffs with PF inhibitor. Scale bar 30 μm. (**f**) Quantification of the number of Iba1 cells. Data are representative of four animals per group and are expressed as mean ± SEM. Data were analyzed using one-way ANOVA with Bonferroni’s post-hoc test: α-syn Mon vs. α-syn pffs, *** *p* < 0.0001; α-syn pffs vs. α-syn pffs with MLi2, *** *p* = 0.0001; α-syn pffs vs. α-syn pffs with PF, *** *p* < 0.0001. (**g**) Quantification of Iba1 immunoreactivity expressed as intensity density. Data are representative of four animals per group and are expressed as mean ± SEM. Data were analyzed using one-way ANOVA with Bonferroni’s post-hoc test: α-syn Mon vs. α-syn pffs, *** *p* = 0.0009; α-syn pffs vs. α-syn pffs with MLi2, ** *p* = 0.0047; α-syn pffs vs. α-syn pffs with PF, * *p* = 0.0128. (**h**) Quantification of Iba1 immunoreactivity expressed as % area occupied by the signal. Data are representative of four animals per group and are expressed as mean ± SEM. Data were analyzed using one-way ANOVA with Bonferroni’s post-hoc test: α-syn Mon vs. α-syn pffs, *** *p* = 0.0009; α-syn pffs vs. α-syn pffs with MLi2, ** *p* = 0.0046; α-syn pffs vs. α-syn pffs with PF, * *p* = 0.0119.

Then, we started investigating whether LRRK2 kinase MLi2 and PF inhibitors affect neuroinflammatory response by evaluating glial activation in our animal model of AD- or PD-related neuroinflammation. Specifically, we analyzed the hippocampal region of mice injected with Aβ_1-42_ fibrils (the region more affected in AD [35] and close to the injection site) and the striatal region of mice injected with α-syn pffs (the injected site and the region affected in PD [36]). We first assessed astrocyte reactivity by staining brain sections with GFAP. As expected, we found increased levels of the number, intensity signal and area occupied by GFAP-positive astrocytic cells in mice injected with Aβ_1-42_ fibrils (Figure 3a–d) and with α-syn pffs (Figure 4a–d) compared to mice injected with monomeric proteins. Interestingly, these effects were attenuated by treatment with the two LRRK2 kinase inhibitors (Figure 3a–d and Figure 4a–d).

In addition to astrogliosis, we evaluated microglia activation using Iba1 staining. Mice injected with Aβ_1-42_ fibrils (Figure 3e–h) and with α-syn pffs (Figure 4e–h) displayed an increment in the number, intensity signal and area occupied by Iba1-positive cells compared to mice injected with monomeric proteins, the effects of which are significantly reduced in the presence of both LRRK2 inhibitors. Taken together, these results indicate that LRRK2 kinase inhibition attenuated the astrocytic and microglial response in the two animal models analyzed.

### 3.3. LRRK2 Inhibition Attenuates Neuroinflammation Induced by Aβ_1-42_ Fibril or α-syn pff Intracerebral Injection

**Figure 5:**
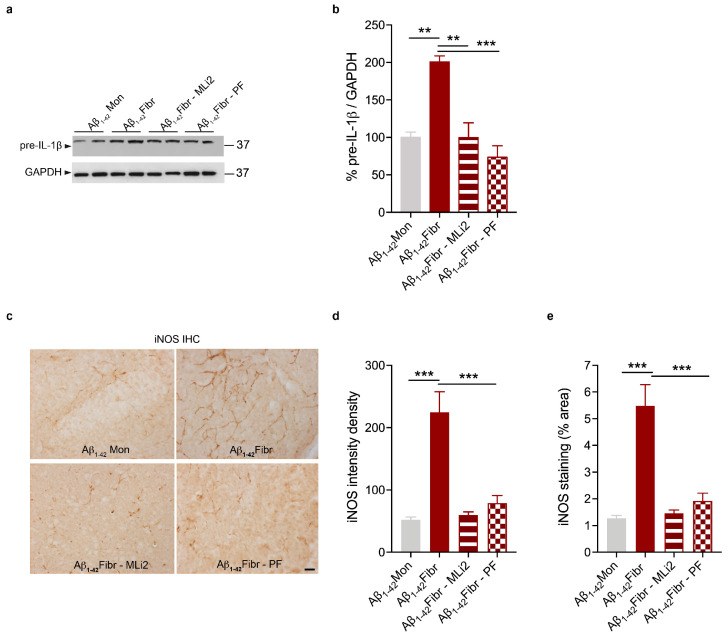
LRRK2 inhibition attenuates Aβ_1-42_ fibril-mediated neuroinflammation. (**a**) Hippocampal tissue lysates from mice injected with Aβ_1-42_ Mon, Aβ_1-42_ fibrils, Aβ_1-42_ fibrils with MLi2, and Aβ_1-42_ fibrils with PF inhibitor were subjected to immunoblotting using IL-1β and GAPDH antibodies. (**b**) Quantification of pre-IL-1β is normalized to GAPDH and expressed as %. Data are representative of at least three animals per group and are expressed the mean ± SEM. Data were analyzed using one-way ANOVA with Bonferroni’s post-hoc test: Aβ_1-42_ Mon vs. Aβ_1-42_ fibrils, ** *p* = 0.0038; Aβ_1-42_ fibrils vs. Aβ_1-42_ fibrils with MLi2, ** *p* = 0.0021; Aβ_1-42_ fibrils vs. Aβ_1-42_ fibrils with PF, *** *p* = 0.0003. (**c**) Representative images of iNOS staining in the hippocampus of mice injected with Aβ_1-42_ Mon, Aβ_1-42_ fibrils, Aβ_1-42_ fibrils with MLi2, and Aβ_1-42_ fibrils with PF inhibitor. Scale bar 30 μm. (**d**) Quantification of iNOS immunoreactivity expressed as intensity density. Data are representative of at least three animals per group and are expressed as mean ± SEM. Data were analyzed using one-way ANOVA with Bonferroni’s post-hoc test: Aβ_1-42_ Mon vs. Aβ_1-42_ fibrils, *** *p* = 0.0004; Aβ_1-42_ fibrils vs. Aβ_1-42_ fibrils with MLi2, *** *p* = 0.0003; Aβ_1-42_ fibrils vs. Aβ_1-42_ fibrils with PF, *** *p* = 0.0009. (**e**) Quantification of iNOS immunoreactivity expressed as % area occupied by the signal. Data are representative of at least three animals per group and are expressed as mean ± SEM. Data were analyzed using one-way ANOVA with Bonferroni’s post-hoc test: Aβ_1-42_ Mon vs. Aβ_1-42_ fibrils, *** *p* = 0.0004; Aβ_1-42_ fibrils vs. Aβ_1-42_ fibrils with MLi2, *** *p* = 0.0003; Aβ_1-42_ fibrils vs. Aβ_1-42_ fibrils with PF, *** *p* = 0.0009.

**Figure 6:**
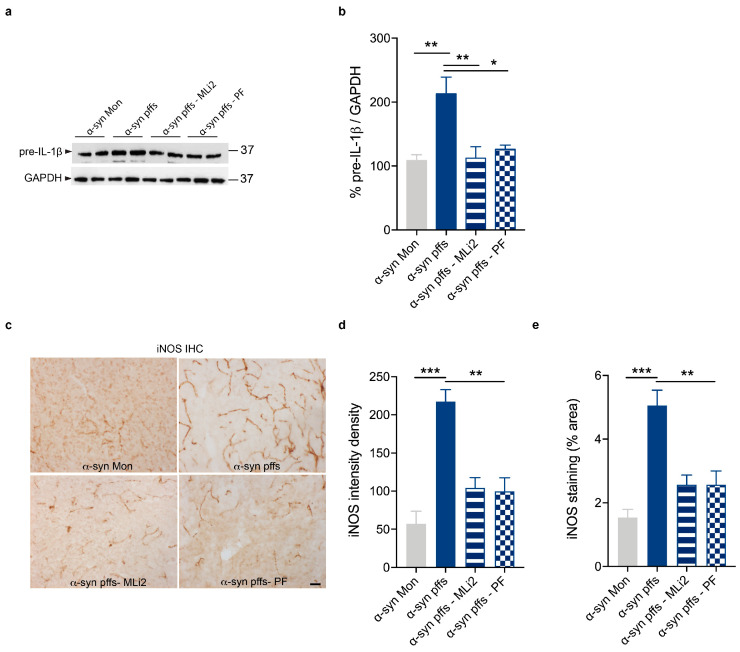
LRRK2 inhibition attenuates α-syn pff-mediated neuroinflammation. (**a**) Striatal tissue lysates from mice injected with α-syn Mon, α-syn pffs, α-syn pffs with MLi2, and α-syn pffs with PF inhibitor were subjected to immunoblotting using IL-1β and GAPDH antibodies. (**b**) Quantification of pre-IL-1β is normalized to GAPDH and expressed as %. Data are representative of four animals per group and are expressed as mean ± SEM. Data were analyzed using one-way ANOVA with Bonferroni’s post-hoc test: α-syn Mon vs. α-syn pffs, ** *p* = 0.0055; α-syn pffs vs. α-syn pffs with MLi2, ** *p* = 0.0073; α-syn pffs vs. α-syn pffs with PF, * *p* = 0.0205. (**c**) Representative images of iNOS staining in the striatum of mice injected with α-syn Mon, α-syn pffs, α-syn pffs with MLi2, and α-syn pffs with PF inhibitor. Scale bar 30 μm. (**d**) Quantification of iNOS immunoreactivity expressed as intensity density. Data are representative of at four animals per group and are expressed as mean ± SEM. Data were analyzed using one-way ANOVA with Bonferroni’s post-hoc test: α-syn Mon vs. α-syn pffs, *** *p* < 0.0001; α-syn pffs vs. α-syn pffs with MLi2, ** *p* = 0.0018; α-syn pffs vs. α-syn pffs with PF, ** *p* = 0.0013. (**e**) Quantification of iNOS immunoreactivity expressed as % area occupied by the signal. Data are representative of four animals per group and are expressed as mean ± SEM. Data were analyzed using one-way ANOVA with Bonferroni’s post-hoc test: α-syn Mon vs. α-syn pffs, *** *p* = 0.0002; α-syn pffs vs. α-syn pffs with MLi2, ** *p* = 0.0035; α-syn pffs vs. α-syn pffs with PF, ** *p* = 0.0035.

We then assessed that LRRK2 kinase inhibition reduces the neuroinflammatory response in our in vivo model of AD- and PD-related neuroinflammation by analyzing the proinflammatory mediators IL-1β and iNOS. As shown in Figure 5 and Figure 6, Aβ_1-42_ fibrils (Figure 5a,b) and α-syn pffs (Figure 6a,b) lead to increased levels of the IL-1 β precursor (pre-IL-1β) compared to mice injected with monomeric proteins, the increment of which is strongly reduced in the presence of both LRRK2 MLi2 and PF inhibitors. In addition to IL-1β, we also evaluated iNOS, which generates nitric oxide (NO) during inflammation and participates in neuronal damage in diseased brains [37,38]. As expected, we found augmented expression of iNOS signal in mice injected with Aβ_1-42_ fibrils (Figure 5c–e) and with α-syn pffs (Figure 6c–e), which is robustly mitigated in the presence of LRRK2 kinase inhibition. These observations confirmed that LRRK2 kinase activity controls the generation of proinflammatory mediators triggered by amyloid proteins and that LRRK2 inhibition could have anti-inflammatory effects in NDDs with an inflammatory component. Moreover, of relevance, our results showed, for the first time in vivo, that LRRK2-mediated neuroinflammation might contribute to AD pathogenesis.

### 3.4. LRRK2 Kinase Inhibition Prevents Aβ_1-42_ Fibril- and α-syn pff-Induced Cell Toxicity

**Figure 7:**
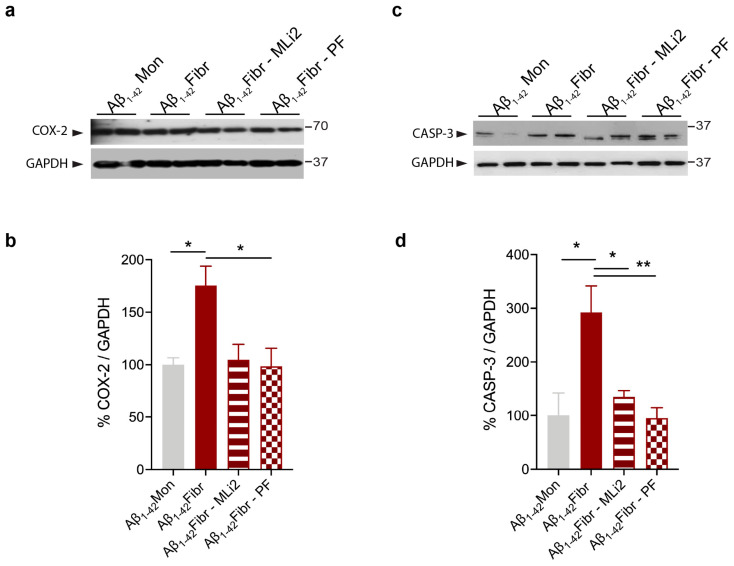
LRRK2 inhibition attenuates Aβ_1-42_ fibril-mediated cell toxicity. (**a**) Hippocampal tissue lysates from mice injected with Aβ_1-42_ Mon, Aβ_1-42_ fibrils, Aβ_1-42_ fibrils with MLi2, and Aβ_1-42_ fibrils with PF inhibitor were subjected to immunoblotting using COX-2 and GAPDH antibodies. (**b**) Quantification of COX-2 is normalized to GAPDH and expressed as %. Data are representative of at least three animals per group and are expressed as mean ± SEM. Data were analyzed using one-way ANOVA with Bonferroni’s post-hoc test: Aβ_1-42_ Mon vs. Aβ_1-42_ fibrils, * *p* = 0.0497; Aβ_1-42_ fibrils vs. Aβ_1-42_ fibrils and MLi2, * *p* = 0.0463; Aβ_1-42_ fibrils vs. Aβ_1-42_ fibrils and PF, * *p* = 0.0279. (**c**) Hippocampal tissue lysates from mice injected with Aβ_1-42_ Mon, Aβ_1-42_ fibrils, Aβ_1-42_ fibrils with MLi2, and Aβ_1-42_ fibrils with PF inhibitor were subjected to immunoblotting using CASP-3 and GAPDH antibodies. (**d**) Quantification of CASP-3 is normalized to GAPDH and expressed as %. Data are representative of at least three animals per group and are expressed as mean ± SEM. Data were analyzed using one-way ANOVA with Bonferroni’s post-hoc test: Aβ_1-42_ Mon vs. Aβ_1-42_ fibrils, * *p* = 0.0155; Aβ_1-42_ fibrils vs. Aβ_1-42_ fibrils and MLi2, * *p* = 0.0340; Aβ_1-42_ fibrils vs. Aβ_1-42_ fibrils and PF, ** *p* = 0.0075.

**Figure 8:**
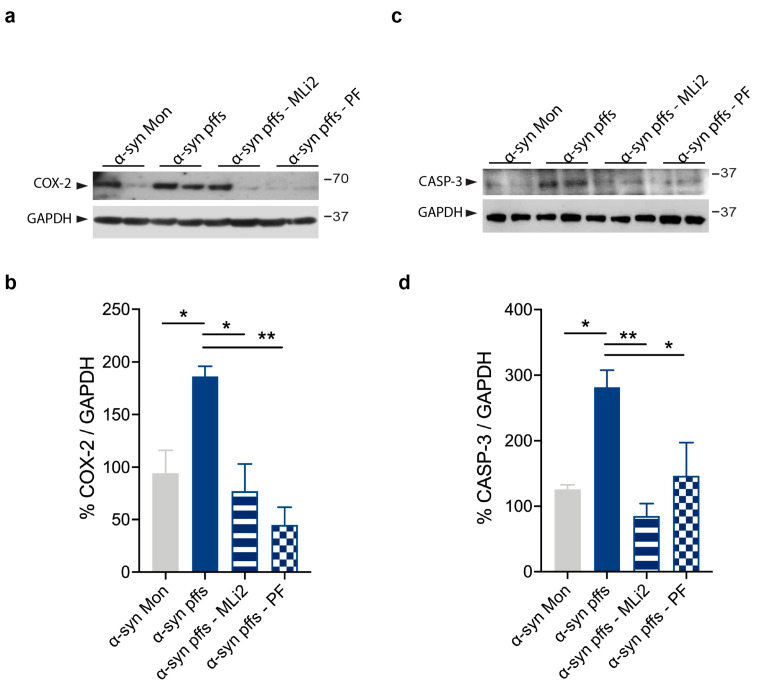
LRRK2 inhibition attenuates α-syn pff-mediated cell toxicity. (**a**) Striatal tissue lysates from mice injected with α-syn Mon, α-syn pffs, α-syn pffs with MLi2, and α-syn pffs with PF inhibitor were subjected to immunoblotting using COX-2 and GAPDH antibodies. (**b**) Quantification of COX-2 is normalized to GAPDH and expressed as %. Data are representative of four animals per group and are expressed as mean ± SEM. Data were analyzed using one-way ANOVA with Bonferroni’s post-hoc test: α-syn Mon vs. α-syn pffs, * *p* = 0.0358; α-syn pffs vs. α-syn pffs with MLi2, * *p* = 0.0117; α-syn pffs vs. α-syn pffs with PF, ** *p* = 0.0015. (**c**) Striatal tissue lysates from mice injected with α-syn Mon, α-syn pffs, α-syn pffs with MLi2, and α-syn pffs with PF inhibitor were subjected to immunoblotting using CASP-3 and GAPDH antibodies. (**d**) Quantification of CASP-3 is normalized to GAPDH and expressed as %. Data are representative of four animals per group and are expressed as mean ± SEM. Data were analyzed using one-way ANOVA with Bonferroni’s post-hoc test: α-syn Mon vs. α-syn pffs, * *p* = 0.0199; α-syn pffs vs. α-syn pffs with MLi2, ** *p* = 0.0036; α-syn pffs vs. α-syn pffs with PF, * *p* = 0.0481.

Previous studies have reported neurotoxicity and degeneration in animal models with Aβ_1-42_ icv injection [39,40]. Thus, we investigated whether LRRK2 kinase inhibition can prevent cell toxicity in our animal models of AD and PD. To this aim, we assessed the induction of COX-2, which is implicated in the cytotoxicity associated with inflammation [41,42], and CASP-3, a proapoptotic marker [39]. We observed increased levels of COX-2 and CASP-3 in the hippocampus of Aβ_1-42_ fibril-injected mice (Figure 7) and in the striatum of the α-syn pff-injected mice compared to their respective control mice (Figure 8). Interestingly, the treatment with both LRRK2 inhibitors significantly attenuated the induction of proapoptotic markers in both animal models (Figure 7 and Figure 8). These results indicate that LRRK2 contributes to cell toxicity in response to Aβ_1-42_ fibril and α-syn pff injection and, importantly, suggest that LRRK2 kinase inhibition could protect cell viability.

## 4. Discussion

LRRK2 is a target with increasing importance for the treatment of NDDs with an inflammatory component; however, further investigation is needed to corroborate its anti-inflammatory properties in preclinical models. In this regard, our study was designed to provide more evidence and elucidate the effect of LRRK2 kinase modulation on neuroinflammation in the context of inflamed brains. Specifically, we explored the effects of LRRK2 MLi2 and PF inhibitors in animal models of AD- and PD-related neuroinflammation. Interestingly, we showed that LRRK2 kinase inhibition significantly reduced neuroinflammation and gliosis, preventing cytotoxicity in mice with intracerebral injection of Aβ_1-42_ fibrils or α-syn pffs. Thus, our findings confirmed that LRRK2 kinase inhibition has anti-inflammatory effects and could be protective and beneficial for brain disorders with an inflammatory component.

Neuroinflammation is a well-described condition in several NDDs, including PD and AD, and widely contributes to the neurodegeneration and progression of the pathology [6,43,44]. In this regard, elevated levels of proinflammatory cytokines have been found in the cerebrospinal fluid and brains of experimental animal models and patients with PD [45,46] and AD [47,48]. Moreover, activated microglia and reactive astrocytes have been reported around dopaminergic neurons in the substantia nigra pars compacta (SNpc) of patients with PD [49], and around amyloid plaques in AD brains [50,51]. Overall, these observations indicate that brain immune cells and their mediators contribute to neuronal dysfunctions and degeneration and, importantly, propose that the modulation of the neuroinflammatory reaction might be a disease-modifying therapeutic strategy for these diseases.

Of relevance, it is well accepted that LRRK2 is a positive mediator of the brain’s immune response and that its modulation might have anti-inflammatory effects and be neuroprotective. Compelling evidence has demonstrated that microglia and astrocyte cultured cells treated with different inhibitory molecules of LRRK2 kinase activity exhibited attenuated inflammation and immune cell-related functions in response to different challenges [16]. As well as in vitro systems, targeting LRRK2-related inflammation has been beneficial even to preclinical models of PD. In this context, a significant reduction in α-syn-mediated neuroinflammation, with attenuated microglial activation and T cell infiltration, in the adeno-associated viral (AAV) vector-based PD model treated with LRRK2 kinase inhibitor MLi2 has been shown [29]. Daher et al. [23] reported that the exacerbated α-syn-induced neuroinflammation and neurodegeneration observed in G2019S-LRRK2 transgenic rats could be mitigated by the treatment of LRRK2 PF inhibitor. Accordingly, the Morari group reported that LRRK2 PF and MLi2 inhibitors protected from 1-methyl-4-phenyl-1,2,5,6-tetrahydropyridine (MPTP)-induced neurotoxicity and gliosis [28]. In addition to the PD-related state, lowering LRRK2 kinase activity has been shown to have anti-inflammatory properties even in a mouse model with spinal cord injury [27], thus supporting the idea that LRRK2 could be targeted and be beneficial for different brain diseases with an inflammatory component. Interestingly, in this study, we provide additional proof of the proinflammatory response’s effects in preclinical models with pathological conditions linked to AD and PD, as well as the ability of LRRK2 kinase inhibition to attenuate them. We showed that the two LRRK2 kinase inhibitors, MLi2 and PF, significantly reduce neuroinflammatory mediators and gliosis, preventing cell toxicity mediated by Aβ_1-42_ fibril and α-syn pff intracerebral injection. Overall, our results add to evidence of the beneficial effect of targeting LRRK2-mediated neuroinflammation in diseased brains.

In addition to inflammation, LRRK2 has been associated with α-syn and β-amyloid even in relation to their pathogenic actions. Specifically, LRRK2 interacts with and phosphorylates amyloid precursor protein (APP) at threonine (Thr) 668, wherein phosphorylation has been implicated in the generation of amyloid deposits in hippocampal neurons of AD brains [52]. Moreover, we recently showed that LRRK2 kinase activity impacts the uptake and clearance of aβ_1-42_ aggregates by astrocytes [11], indicating that LRRK2 may affect β-amyloid pathology at the level of both neurons and glia. However, in support of LRRK2 and α-syn interplay, studies suggest that LRRK2 is involved in the aggregation and spreading of α-syn through the regulation of the endolysosomal pathways in both neuron and glia [53,54,55]. Taken together, these observations indicate that LRRK2 might be implicated in the pathogenesis of AD and PD in multiple ways and, importantly, suggest that targeting LRRK2 kinase activity could also be beneficial to reducing the spread of these diseases.

Intriguingly, for the first time, we reported that LRRK2 kinase activity contributes to AD-related neuroinflammation in vivo. In accordance with these findings, we recently showed that LRRK2 pharmacological inhibition attenuates Aβ_1-42_-induced astrocytic inflammation and favors the clearance of Aβ_1-42_ fibrils in cultured cells [11], thus linking LRRK2-mediated inflammation to AD pathology. Neuroinflammation is involved in different aspects of AD and plays a crucial role in the progression of the disease. In this regard, it has been reported that proinflammatory mediators might potentiate the enzymatic activity of Tau kinases and γ- and β-secretases, enhancing the deposition of intracellular phosphorylated Tau [56] and amyloid-β accumulation [57,58], respectively. Overall, these observations support a key contribution of neuroinflammation in promoting AD pathogenesis and, importantly, suggest that LRRK2, which is a positive mediator of neuroinflammation, could mediate and contribute to AD pathogenesis. Certainly, more investigations are required to shed light on the contribution of PD-linked LRRK2 to AD pathogenesis.

## 5. Conclusions

Overall, our study corroborates the anti-inflammatory properties of LRRK2 kinase inhibition in preclinical models of AD- and PD-related neuroinflammation and supports the hypothesis that targeting LRRK2 activity could be protective and beneficial for brain disorders with an inflammatory component.

## Data Availability

The datasets supporting the conclusion of this article are available in the ZENODO repository (10.5281/zenodo.8059360) from the corresponding author upon reasonable request.

## Supplementary Materials

The following supporting information can be downloaded at: https://www.mdpi.com/article/10.3390/cells12131799/s1, Figure S1. Evaluation of Ser935-LRRK2 phosphorylation in the brain of mice.
