# Supplementary material for: LRRK2 Kinase Inhibition Attenuates Neuroinflammation and Cytotoxicity in Animal Models of Alzheimer’s and Parkinson’s Disease-Related Neuroinflammation
Source: Cells. 2023 Jul 6;12(13):1799. doi: 10.3390/cells12131799 (PMC10340668; doi:10.3390/cells12131799)
Supplement: Supplementary file 1 [file cells-12-01799-s001.zip › cells-2331723-supplementary.pdf]

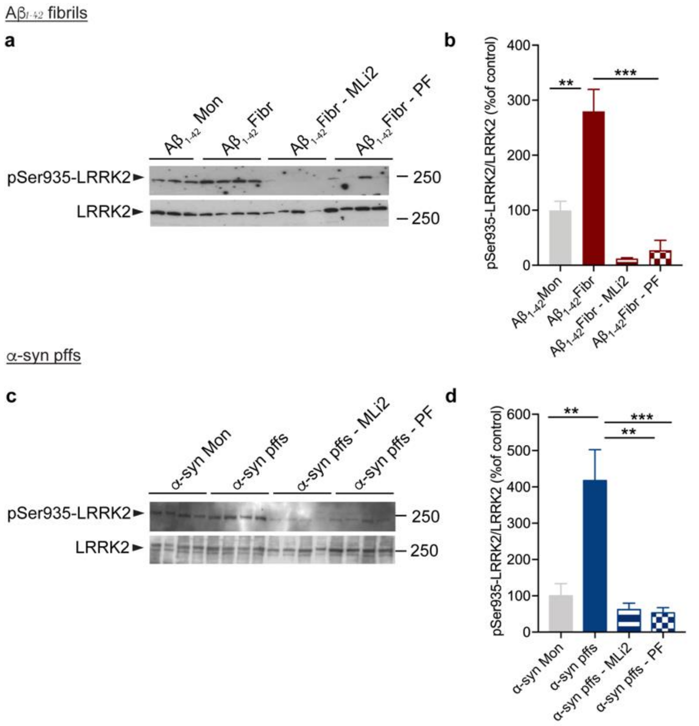

Supplementary Figure S1: Evaluation of Ser935-LRRK2 phosphorylation in the brain of mice. Mice received LRRK2 inhibitors PF and MLi2 (10 mg/kg, twice daily, ip) or vehicle (twice daily, ip) for 10 days. 3 Days after the initiation of drug administration, amyloid proteins (Aβ$_{1-42}$ or α-syn aggregated or monomeric proteins) were intracerebrally injected. Mice were then sacrificed at 1 week after the intracerebral injections. **(a)** Tissue lysates from cortex of mice injected with Aβ$_{1-42}$ Mon, Aβ$_{1-42}$ fibrils, Aβ$_{1-42}$ fibrils with MLi2 inhibitor, and Aβ$_{1-42}$ fibrils with PF inhibitor were subjected to immunoblotting using pSer935-LRRK2 and LRRK2 antibodies. **(b)** Quantification of pSer935-LRRK2 is normalized to total LRRK2 and expressed as %. Data are representative of at least three animals per group and are expressed as the mean ± SEM. Data were analyzed using One-Way ANOVA with Bonferroni's post-hoc test: Aβ$_{1-42}$ Mon *vs.* Aβ$_{1-42}$ fibrils, **p =0.0025; Aβ$_{1-42}$ fibrils *vs.* Aβ$_{1-42}$ fibrils with MLi2, ***p<0.0001; Aβ$_{1-42}$ fibrils *vs.* Aβ$_{1-42}$ fibrils with PF, ***p<0.0001. **(c)** Tissue lysates from cortex of mice injected with α-syn Mon, α-syn pffs, α-syn pffs with MLi2, and α-syn pffs with PF inhibitor were subjected to immunoblotting using pSer935-LRRK2 and LRRK2 antibodies. **(d)** Quantification of pSer935-LRRK2 is normalized to total LRRK2 and expressed as %. Data are representative of four animals per group and are expressed as the mean ± SEM. Data were analyzed using One-Way ANOVA with Bonferroni's post-hoc test: α-syn Mon *vs.* α-syn pffs, **p=0.0028; α-syn pffs *vs.* α-syn pffs with MLi2, **p=0.0011; α-syn pffs *vs.* α-syn pffs with PF, ***p=0.0009.
